# Classification of amyloid status using machine learning with histograms of oriented 3D gradients

**DOI:** 10.1016/j.nicl.2016.05.004

**Published:** 2016-05-10

**Authors:** Liam Cattell, Günther Platsch, Richie Pfeiffer, Jérôme Declerck, Julia A. Schnabel, Chloe Hutton, for the Alzheimer's Disease Neuroimaging Initiative

**Affiliations:** a Institute of Biomedical Engineering, Department of Engineering Science, University of Oxford, UK; b Siemens Molecular Imaging, Oxford, UK; c Piramal Imaging, Berlin, Germany; d Division of Imaging Sciences and Biomedical Engineering, King's College London, UK

**Keywords:** Amyloid, Positron emission tomography, Florbetapir, Florbetaben, Pittsburgh compound B, Classification

## Abstract

Brain amyloid burden may be quantitatively assessed from positron emission tomography imaging using standardised uptake value ratios. Using these ratios as an adjunct to visual image assessment has been shown to improve inter-reader reliability, however, the amyloid positivity threshold is dependent on the tracer and specific image regions used to calculate the uptake ratio. To address this problem, we propose a machine learning approach to amyloid status classification, which is independent of tracer and does not require a specific set of regions of interest. Our method extracts feature vectors from amyloid images, which are based on histograms of oriented three-dimensional gradients. We optimised our method on 133 ^18^F-florbetapir brain volumes, and applied it to a separate test set of 131 volumes. Using the same parameter settings, we then applied our method to 209 ^11^C-PiB images and 128 ^18^F-florbetaben images. We compared our method to classification results achieved using two other methods: standardised uptake value ratios and a machine learning method based on voxel intensities. Our method resulted in the largest mean distances between the subjects and the classification boundary, suggesting that it is less likely to make low-confidence classification decisions. Moreover, our method obtained the highest classification accuracy for all three tracers, and consistently achieved above 96% accuracy.

## Highlights

• A machine learning approach to brain amyloid status classification is proposed.
• The method is independent of PET tracer and requires little recalibration.
• Classification accuracy was higher than SUVR for three amyloid tracers.

## 1 Introduction

Positron emission tomography (PET) is increasingly used to assess the burden of fibrillar *β*-amyloid in patients with suspected Alzheimer's disease (AD). Elevated levels of *β*-amyloid, in the form of plaques, are a pathological biomarker of the disease. The first tracer to specifically image these plaques in neuronal tissue was ^11^C-Pittsburgh Compound-B (^11^C-PiB) (Klunk et al., 2004). Due to the short half-life of carbon-11 (20 min), the compound needs to be prepared on-site and used immediately. This requires a cyclotron in the hospital, which is uncommon, and hence makes ^11^C-PiB impractical for routine clinical use. More recently, several other amyloid tracers have been developed using the fluorine-18 isotope, which has a longer half-life of 110 min and allows regional distribution. Three of these have recently been approved by the US Food and Drug Administration (FDA) for use in clinical diagnosis: ^18^F-florbetapir, ^18^F-flutemetamol, and ^18^F-florbetaben (FDA, 2012, FDA, 2013, Piramal, 2014).

Prior to regulatory approval, the FDA and European Medicines Association (EMA) gave much attention to consistency of F-18 amyloid image interpretation between readers (EMA Committee for Medicinal Products for Human Use, 2013, FDA Peripheral and Central Nervous System Drugs Advisory Co). Consequently, thorough reader training programmes have been developed for visual interpretation. However, as reported by Frey (2015), a lack of concordance between independent readers suggests the need for additional analytical approaches to clinical reading and reporting.

Brain amyloid burden can be evaluated quantitatively by calculating the ratio of tracer uptake in a set of target brain regions to non-specific tracer uptake in a reference region (Barthel et al., 2011, Fleisher et al., 2011, Jack et al., 2008, Jagust et al., 2009, Joshi et al., 2012, Villemagne et al., 2011). This ratio is known as the standardised uptake value ratio (SUVR). Typically, the individual SUVRs for each target region are averaged to form the mean, or *composite*, SUVR (Rowe et al., 2008). It has been shown that incorporating this ratio as an adjunct to the visual assessment of ^18^F-florbetapir scans can decrease inter-reader variability (Nayate et al., 2015, Pontecorvo et al., 2014).

The composite SUVR is usually used in a discrete fashion, where subjects above a particular threshold are designated as amyloid positive, and subjects below a certain threshold are designated as amyloid negative (Landau et al., 2013). The thresholds are dependent on the type of tracer, the brain regions used to calculate the composite SUVR, and the delineation of those regions. Examples of amyloid positivity thresholds and SUVR target and reference regions are shown in Table 1. Although there is a consensus in the literature about which general brain areas are to be used in the composite SUVR, there are differences in the details (Jack et al., 2008, Jagust et al., 2009). In practice, it means that for a specific tracer, the correct set of regions and thresholds must be known and applied. For example, since amyloid deposition does not typically occur in the cerebellum, the reference regions presented in Table 1 are all cerebellum-based. Nevertheless, Jack et al. (2008) use the cerebellar grey matter, whereas Joshi et al. (2012) use the whole cerebellum. Moreover, the frontal lobe is a universal target region, but the delineation of the region varies with the study; Barthel et al. (2011) use the frontal lobe, whereas Villemagne et al. (2011) use specific areas within the frontal lobe.

A method for amyloid status classification, independent of SUVR, was proposed by Vandenberghe et al. (2013). The authors classified ^18^F-flutemetamol scans as amyloid positive or amyloid negative using a machine learning method known as a support vector machine (SVM) (Cortes and Vapnik, 1995). The SVM was trained using the voxel intensities, and the leave-one-out testing method achieved 100% agreement with the visual image assessments.

In this work, we propose an alternative machine learning method, which could serve as an adjunct to visual image interpretation, like composite SUVR, but without the need for defining tracer-specific regions of interest and selecting positivity thresholds. Our method trains an SVM using features based on histograms of oriented 3D gradients (3D HOG) rather than using image intensity directly. The aim of this work is therefore to compare the accuracy of amyloid status classification obtained using our new method (3D HOG + SVM) with the intensity-based SVM, and with the standard approach based on SUVR. We show that our method can be used across a range of amyloid tracers, without the need to define different brain regions or positivity thresholds; we trained our method using 133 ^18^F-florbetapir images and applied it directly to 209 ^11^C-PiB images and 128 ^18^F-florbetaben images with favourable results.

The rest of this paper is structured as follows: in Section 2 we present an overview of the data and the preprocessing steps. Section 2 also introduces our proposed method of combining 3D HOG with an SVM, and reviews both the intensity-based SVM and SUVR methods. In Section 3, we present the results for the three classification methods, as well as detailing the results of the 3D HOG optimisation process. Finally, in Section 4, we discuss the advantages of our proposed method over the other two classification methods, and conclude this work.

## 2 Materials and methods

### 2.1 Alzheimer's Disease Neuroimaging Initiative (ADNI) data

Data used in the preparation of this article were obtained from the ADNI database (adni.loni.usc.edu). The ADNI was launched in 2003 by the National Institute on Aging (NIA), the National Institute of Biomedical Imaging and Bioengineering (NIBIB), the US (FDA), private pharmaceutical companies and non-profit organisations, as a $60 million, 5-year public–private partnership. The primary goal of ADNI has been to test whether serial magnetic resonance imaging (MRI), PET, other biological markers, and clinical and neuropsychological assessment can be combined to measure the progression of mild cognitive impairment (MCI) and early AD. Determination of sensitive and specific markers of very early AD progression is intended to aid researchers and clinicians to develop new treatments and monitor their effectiveness, as well as lessen the time and cost of clinical trials.

The Principal Investigator of this initiative is Michael W. Weiner, M.D., VA Medical Center and University of California, San Francisco. ADNI is the result of efforts of many coinvestigators from a broad range of academic institutions and private corporations, and subjects have been recruited from over 50 sites across the USA and Canada. The initial goal of ADNI was to recruit 800 subjects but ADNI has been followed by ADNI-GO and ADNI-2. To date these three protocols have recruited over 1500 adults, aged 55–90, to participate in the research, consisting of cognitively normal older individuals, people with early or late MCI and people with early AD. The follow-up duration of each group is specified in the protocols for ADNI-1, ADNI-2 and ADNI-GO. Subjects originally recruited for ADNI-1 and ADNI-GO had the option to be followed in ADNI-2. For up-to-date information, see www.adni-info.org.

### 2.2 Data acquisition and pre-processing

**Fig. 1:**
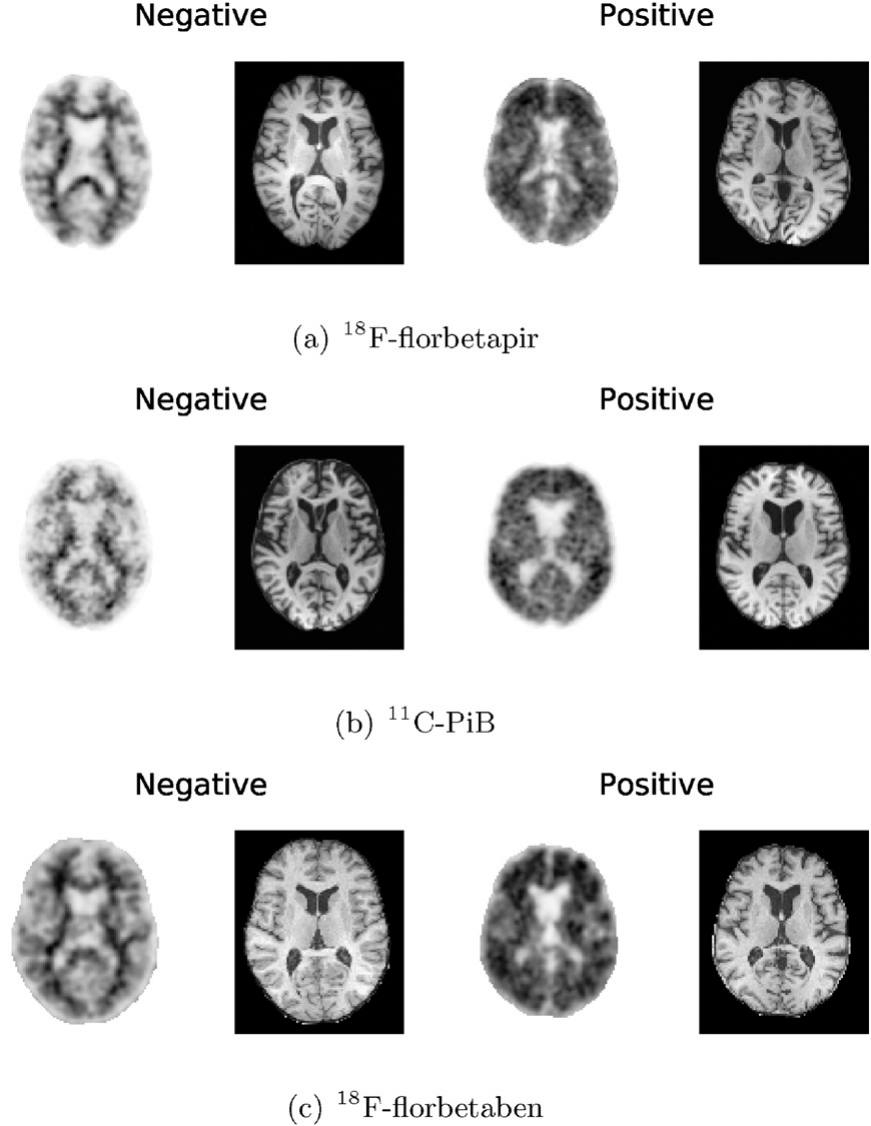
Axial slices from example amyloid negative (left) and amyloid positive (right) PET volumes for (a) ^18^F-florbetapir, (b) ^11^C-PiB, and (c) ^18^F-florbetaben. The corresponding axial MR slices are shown to the right of each PET image. These slices were selected after the PET and MR volumes had been preprocessed.

^18^F-florbetapir PET and T1-weighted MR volumes from 294 subjects were gathered from the ADNI database. The two volumes for each subject selected for this study were acquired no more than 12 months apart. Although the scans were acquired at multiple sites, all sites followed the same ADNI protocol.^2^ For the purpose of this work, the ^18^F-florbetapir PET volumes were rigidly registered to their corresponding MR volumes using Statistical Parametric Mapping, version 8^3^ (SPM8). The MR volumes were then affinely registered to Montreal Neurological Institute (MNI) space using FSL's FLIRT software (Jenkinson et al., 2002, Jenkinson and Smith, 2001), and the resulting transformations were applied to the ^18^F-florbetapir volumes. Finally, the MR and ^18^F-florbetapir images were skull-stripped using a brain mask constructed from MR tissue segmentations obtained using SPM8. Prior to the classification experiments, the transformed ^18^F-florbetapir PET brain volumes were resampled to 2 × 2 × 2 mm resolution. Axial slices from examples of amyloid negative and amyloid positive ^18^F-florbetapir brain volumes, along with their corresponding MR slices, are shown in Fig. 1(a).

In addition to the ^18^F-florbetapir data, 214 ^11^C-PiB and corresponding T1-weighted MR volumes were downloaded from the ADNI database. The data belonged to 102 subjects, but even though some subjects had multiple scans (26 subjects had one scan, 42 subjects had two scans, 32 subjects had three scans, and two subjects had four scans), each ^11^C-PiB/MR pair was treated independently. Each ^11^C-PiB volume and its corresponding MR volume were acquired within 12 months of one another. Furthermore, the ^11^C-PiB scans underwent the same pre-processing as the ^18^F-florbetapir volumes. Example amyloid positive and amyloid negative axial slices from the ^11^C-PiB dataset are shown in Fig. 1(b).

The ^18^F-florbetaben PET volumes and corresponding T1-weighted MR volumes were provided from the phase 2A clinical trial of ^18^F-florbetaben in 150 participants. The participant details and imaging protocols are all provided in Barthel et al. (2011). Seventeen subjects were excluded due to severe image artefacts in the PET or MR images. The MR and ^18^F-florbetaben PET volumes were coregistered using an in-house rigid registration algorithm. The ^18^F-florbetaben images were then registered to a PET template in MNI space using an in-house affine registration algorithm and resampled to 2 × 2 × 2 mm resolution. The in-house rigid and affine registration algorithms were implemented using routines customised from Siemens syngo.PET Amyloid Plaque (sPAP) quantification software. The resulting transformations were applied to the MR volumes. Analogously to the ^18^F-florbetapir and ^11^C-PiB, a brain mask was constructed using tissue segmentations obtained from SPM8. Both the ^18^F-florbetaben PET volumes and their corresponding MR volumes were skull-stripped. Fig. 1(c) shows an example axial slice for a ^18^F-florbetaben amyloid negative and ^18^F-florbetaben amyloid positive brain volume. The corresponding axial MR slices are also shown in Fig. 1(c).

### 2.3 Visual assessment

In this study, the gold standard amyloid status was determined for each subject using criteria based on visual assessments from three image readers. Data were excluded from the study if the median rating was neither amyloid positive nor amyloid negative. To interpret the PET volumes as amyloid positive or amyloid negative, the three image readers (one clinical expert, one senior neuro-PET researcher, and one junior PET image analysis researcher) interpreted the images a total of six times. The junior researcher assessed all of the images three times, the senior neuro-PET researcher interpreted all the images twice, and the clinical expert read all of the images once. The tracers were assessed one at a time (i.e. all ^18^F-florbetapir scans were interpreted before the ^11^C-PiB scans), but for each tracer the images were presented in a random order to prevent observer memory affecting the assessments. Each reader was given instructions on how to display and interpret the images on a set of prearranged slices, without access to the corresponding MR image. For the ^18^F-florbetapir and ^18^F-florbetaben scans, the instructions were based on those provided by the tracer manufacturers (Amyvid, 2012, NeuraCeq, 2014). The only major difference was the addition of an “equivocal” image class, for images that did not clearly fulfil the definitions of positive or negative scans. Note that since the reading instructions were very thorough, “equivocal” was typically only selected when image quality was particularly poor. The instructions for visual assessment of ^11^C-PiB were based on a combination of those by Suotunen et al. (2010) and Cohen et al. (2013). Again, an equivocal image class was included for images that could not be designated as either amyloid positive or amyloid negative.

Using Fleiss' kappa to assess the inter-reader reliability, the six image interpretations showed substantial agreement for all tracers (^18^F-florbetapir: *κ* = 0.71, ^11^C-PiB: *κ* = 0.81, ^18^F-florbetaben: *κ* = 0.84). The gold standard amyloid status was determined from the median of the six image interpretations. Any images for which the median interpretation was not amyloid positive or amyloid negative were discarded. In total, 30 ^18^F-florbetapir images, five ^11^C-PiB images, and five ^18^F-florbetaben images were discarded. The final ^18^F-florbetapir dataset comprised 264 subjects, the final ^11^C-PiB dataset consisted of 209 subjects, and the final ^18^F-florbetaben dataset contained 128 subjects. The demographics of these are summarised in Table 2. It should be noted that although the inter-reader agreement and number of equivocal scans varies by tracer, this is not a reflection of the tracers themselves. The discrepancies are predominantly due to differences in image quality and the distributions of amyloid burden.

### 2.4 Image analysis

In this work we compared three separate amyloid classification methods: our method, which uses histograms of oriented three-dimensional gradients as inputs to an SVM (3D HOG + SVM), another SVM-based method using image intensity directly, and SUVR. The three methods are outlined in 2.5, 2.6, 2.7.

### 2.5 Histogram of oriented 3D gradients (3D HOG)

#### 2.5.1 Derivation of feature vectors

**Fig. 2:**
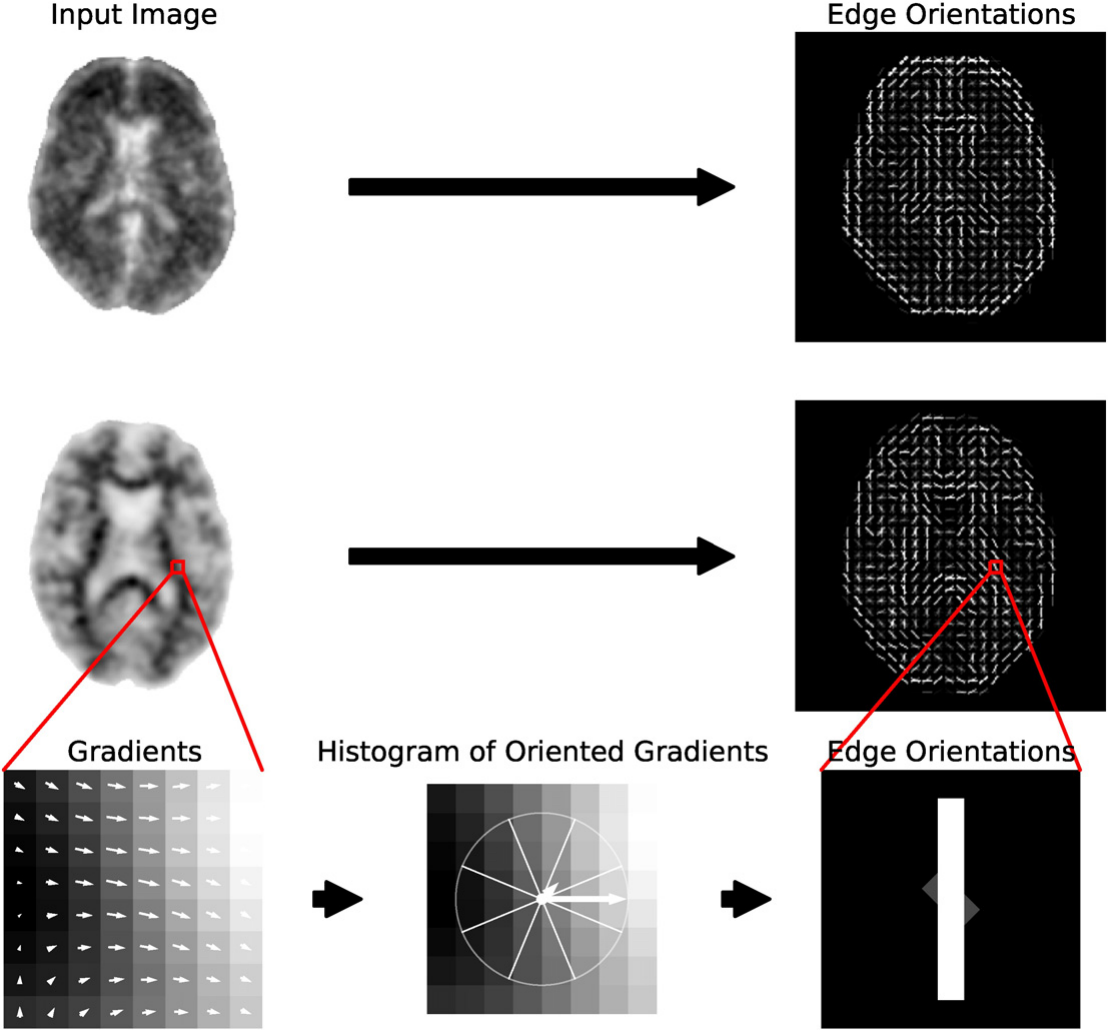
An illustration of 2D HOG features for an amyloid positive ^18^F-florbetapir axial slice (top) and an amyloid negative ^18^F-florbetapir axial slice (middle). The bottom row shows the general steps of the HOG algorithm: image gradients in a single cell (left), quantisation of those gradients (centre), and the edge orientations associated with the histogram of gradients (right). The intensity of edge orientations are determined from the magnitudes of the histogram bins. The actual HOG features used in this work were computed in 3D, as described in Section 2.5.

Image descriptors have been widely used in computer vision to describe characteristics such as texture, motion, and shapes in images and video sequences (Belongie and Malik, 2000, Dalal and Triggs, 2005, Lowe, 1999). Given a region of interest, a descriptor represents the region as a feature vector. By applying machine learning techniques to these feature vectors, they can be used to detect objects in images. One such method, histogram of oriented gradients (HOG), has been used successfully to detect pedestrians in static images (Dalal and Triggs, 2005). The image is partitioned into a grid of uniformly spaced cells, and the normalised histogram of image gradient orientations in each cell forms the set of feature vectors. An illustration of HOG in two dimensions is shown in Fig. 2. The key concepts of this two-dimensional method were generalised to three dimensions by Kläser et al. (2008). Although originally used for action recognition in video volumes, we here propose to apply a similar technique to PET volumes to classify brain amyloid status.

In order to compute histograms of oriented gradients across a PET volume, the volume is partitioned into a uniform grid of cells **c**_*i*_ of size *k* × *k* × *k* voxels. Each cell is divided into *S* × *S* × *S* sub-blocks **b**_*j*_, and for each sub-block the mean gradient is computed. In the same manner as Kläser et al. (2008), we calculated the mean gradient using a 3D extension of the integral image (also known as a summed area table), popularised by Viola and Jones (2001). Given a volume *v*(*x*, *y*, *z*) and its gradient $\text{∇}v={\left(\frac{\text{∂}v}{\text{∂}x},\frac{\text{∂}v}{\text{∂}y},\frac{\text{∂}v}{\text{∂}z}\right)}^{T}$, the integral volume can be written as:

(1) $$I\left(x,y,z\right)=\sum_{x^{\prime }\leq x,y^{\prime }\leq y,z^{\prime }\leq z}\nabla v\left(x^{\prime },y^{\prime },z^{\prime }\right).$$

The mean 3D gradient $\overset{̅}{g}={\left({\overset{̅}{g}}_{x},{\overset{̅}{g}}_{y},{\overset{̅}{g}}_{z}\right)}^{T}$ within a cuboid of size *w* × *h* × *d* at position (*x*, *y*, *z*)^*T*^ is then given by:

(2) $$\begin{array}{l} \overset{̅}{g}=\left(I\left(x+w,y+h,z+d\right)-I\left(x,y+h,z+d\right)\right)\\ \left(\;-I\left(x+w,y,z+d\right)+I\left(x,y,z+d\right)\right)\\ \;-\left(I\left(x+w,y+h,z\right)-I\left(x,y+h,z\right)\right)\\ \;\left(-I\left(x+w,y,z\right)+I\left(x,y,z\right)\right). \end{array}$$

**Fig. 3:**
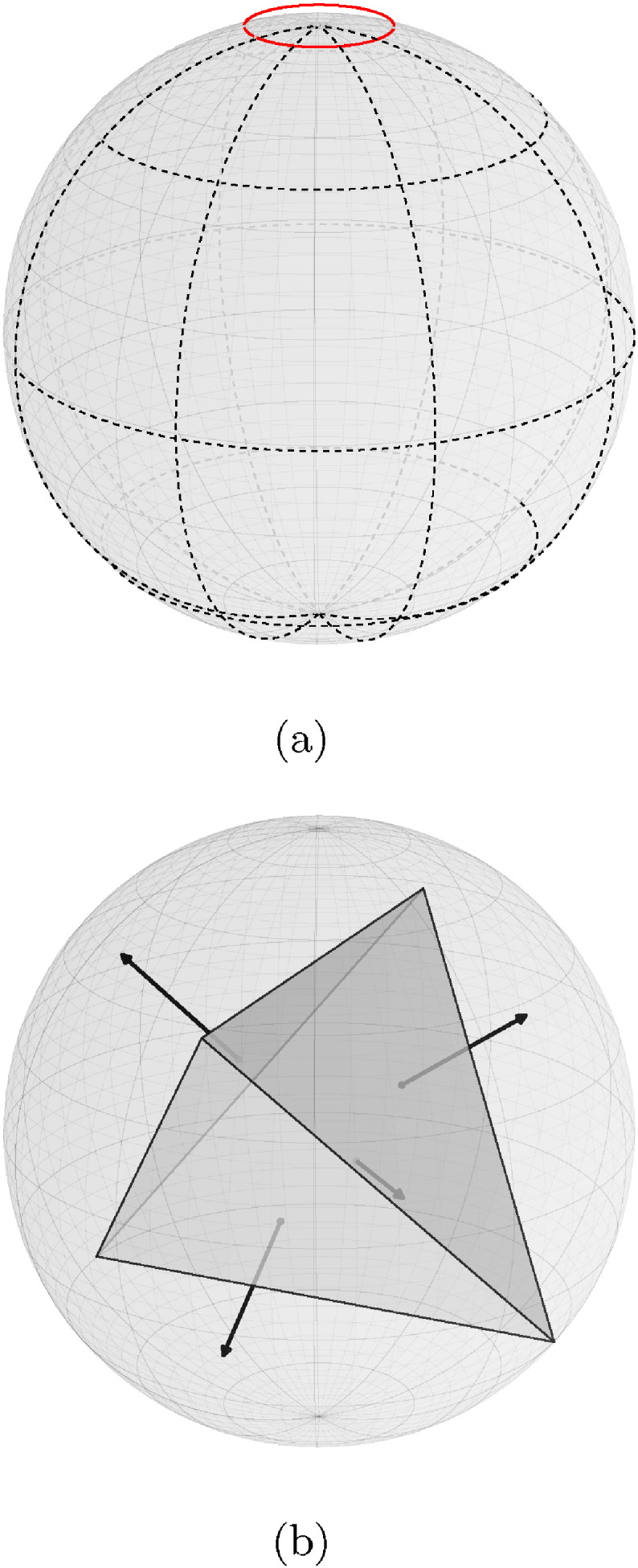
(a) Using spherical polar coordinates to quantise the 3D gradients leads to problems at the poles (red circle) because the bins get progressively smaller. (b) Therefore, we used a regular polyhedron as an approximation to a sphere (Kläser et al., 2008). The 3D gradients are projected on to the vectors from the centre of the polyhedron to the centres of the faces.

Following computation of the 3D gradient $\overset{̅}{g}$, its orientation is quantised into a histogram with *n* discrete bins. A logical extension of the 2D HOG method would be to use spherical polar coordinates to quantise the 3D gradient orientations. By dividing the elevation angle and azimuth into equally sized bins, gradients are quantised using a similar system to latitude and longitude. However, this leads to problems at the poles because the bins get progressively smaller. This is demonstrated by the red circle in Fig. 3(a).

We adopted the solution employed by Kläser et al. (2008), which used a regular polyhedron as an approximation to a sphere. Rather than have a continuous space of orientations, each side of the polyhedron corresponds to a histogram bin. In 3D space, there are only five polyhedra constructed from congruent regular polygons with the same number of faces meeting at each vertex. These polyhedra are known as Platonic solids: tetrahedron, hexahedron (cube), octahedron, dodecahedron, and icosahedron. They have 4, 6, 8, 12, and 20 faces, respectively.

To quantise a 3D gradient $\overset{̅}{g}$ with respect to its orientation, $\overset{̅}{g}$ is projected on to the axes going through the origin of the coordinate system and the centre of all faces of the polyhedron. Letting *P* be the matrix of face centre coordinates **p**_1_ , … , **p**_*n*_, the projection $\hat{q}$ of $\overset{̅}{g}$ is:

(3) $$\hat{q}=\frac{P\cdot \overset{̅}{g}}{{\left\|\overset{̅}{g}\right\|}_{2}}.$$

Opposite gradient directions can be quantised into the same histogram bin by halving the set of face centre coordinates and taking the absolute value of $\hat{q}$. Histograms organised in this manner are said to have “half-orientation”.

Since $\overset{̅}{g}$ should only vote in one histogram bin, the projection $\hat{q}$ is thresholded. The threshold *t* = **p**_*i*_^*T*^ ⋅ **p**_*j*_ is subtracted from $\hat{q}$ and all negative elements are set to zero. The magnitude of the gradient is distributed according to the thresholded projection ${\hat{q}}^{\prime }$:

(4) $$q=\frac{{\left\|\overset{̅}{g}\right\|}_{2}\cdot {\hat{q}}^{\prime }}{{\left\|{\hat{q}}^{\prime }\right\|}_{2}}.$$

The histogram **h**_**c**_*i*__ for a given cell **c**_*i*_ is the sum of the quantised mean gradients of the sub-blocks **q**_**b**_*j*__ in that cell:

(5) $$h_{c_{i}}=\sum_{j=1}^{S^{3}}q_{b_{j}}.$$

The histograms **h**_**c**_*i*__ for each cell are concatenated over the *S*^3^ sub-blocks to form the final feature vector for the entire volume.

#### 2.5.2 Classification

A classifier is required to separate the feature vectors associated with different image classes (e.g. amyloid positive and amyloid negative). Typically a support vector machine (SVM) is used to classify HOG features (Dalal and Triggs, 2005, Kläser et al., 2008). Using a set of correctly labelled data, the SVM tries to find the hyperplane that maximises the margin between the two classes. This hyperplane can then be used to classify previously unseen data (often called test data). Points on one side of the hyperplane are classified as one class, and points on the other side of the hyperplane belong to the other class. In this work we used the SVM implementation in the scikit-learn package for Python (Pedregosa et al., 2011).

#### 2.5.3 Parameter optimisation

In order to determine the optimum parameters for the 3D HOG method, the ^18^F-florbetapir dataset was split into a training and test set. The training set comprised 133 subjects (75 positive, 58 negative), and the test set consisted of 131 subjects (74 positive, 57 negative). All of the ^18^F-florbetapir classification results reported in Section 3 were generated using the test set only. To assess the generalisability of the method, no 3D HOG parameter optimisation was conducted using the ^11^C-PiB and ^18^F-florbetaben data, so the entire datasets were used for testing.

To optimise the parameters for the 3D HOG feature descriptors, we computed feature vectors from the ^18^F-florbetapir training set volumes for a range of parameter values. Cell size ranged from *k* = 4 voxels to *k* = 32 voxels in increments of 4 voxels, and the number of sub-blocks *S* were in the set *S* = {1, 2, 4}. We also assessed the number of histograms bins (dodecahedron and icosahedron), and the effect of full- and half-orientation. A comprehensive grid search of parameters was conducted, resulting in 96 different parameter combinations.

For each of the 3D HOG parameter combinations, a SVM classifier was trained using the corresponding feature vectors of the ^18^F-florbetapir training set. In order to ascertain the optimum SVM parameters to use on the test data, we performed ten-fold stratified cross-validation on the training set. The training set was randomly divided in to 10 subsets, each with the same proportion of amyloid positive/negative subjects. Nine subsets were used to train the SVM, and the remaining subset was used as the validation test dataset. This was repeated, such that each subset was used as the test set. We used a SVM with a Gaussian radial basis function (RBF) kernel, and we optimised the slackness variable *C* (where *C* = 10^*i*^ for *i* = {− 2, … , 3}) and the free parameter of the RBF *γ* (where *γ* = 10^*i*^ for *i* = {− 5, … , 2}) (Boser et al., 1992, Cortes and Vapnik, 1995) using a grid search of parameters.

#### 2.5.4 Testing

The 3D HOG parameters and SVM parameters that gave the highest classification accuracy, sensitivity, and specificity on the ^18^F-florbetapir training set were applied to the ^18^F-florbetapir test set. These parameter values were also applied to the ^11^C-PiB and ^18^F-florbetaben data. Leave-one-out testing was used to assess the performance of the 3D HOG features for amyloid status classification. For each fold, the SVM was trained using all of the subjects except one. The remaining subject was then used as the test subject. This process was repeated until all of the subjects had been used as the test subject. Following leave-one-out testing for all three tracers, we calculated the mean classification accuracy, sensitivity, and specificity. By adjusting the SVM classifier's decision boundary, receiver operating characteristic (ROC) analysis was performed on each of the tracers.

### 2.6 Standardised uptake value ratio

#### 2.6.1 Quantification software

The ratio of tracer uptake in a set of target brain regions to non-specific tracer uptake in a reference region, also known as SUVR, was computed using the commercially available Siemens syngo.PET Amyloid Plaque (sPAP) quantification software. Prior to SUVR calculation, the software automatically registers the subject's PET volume to a synthetic PET template, in MNI space, in which the cortical regions of interest are defined (Hutton et al., 2015, Peyrat et al., 2012). The predefined set of six target regions for ^18^F-florbetapir were: the frontal, parietal, anterior cingulate, posterior cingulate, precuneus, and temporal lobes (Hutton et al., 2015). The reference region was the whole cerebellum. For ^18^F-florbetaben, slightly different predefined target and reference regions were used (target regions: frontal, parietal, anterior cingulate, posterior cingulate, temporal, occipital lobes, reference: cerebellar cortex; Barthel et al., 2011). Note that the different tracers used different sets of regions according to the published literature (Hutton et al., 2015, Barthel et al., 2011, respectively).

The sPAP quantification method has been validated for use with ^18^F-florbetapir and ^18^F-florbetaben (Hutton et al., 2014, Hutton et al., 2015, Peyrat et al., 2012), but not for ^11^C-PiB because it is not an FDA-approved tracer. However, it was still possible to use sPAP for quantification of the ^11^C-PiB data. Based on the literature by Jagust et al. (2009) and Landau et al. (2013), we calculated SUVRs using the ^18^F-florbetapir target and reference regions.

During SUVR computation in sPAP, one ^18^F-florbetapir volume, eight ^18^F-florbetaben volumes, and three ^11^C-PiB volumes failed to adequately register to the PET template. As a result, the registration was manually adjusted for these subjects.

The composite SUVRs calculated using the sPAP software were within the ranges reported in the literature (Barthel et al., 2011, Fleisher et al., 2011). For all three tracers, the mean composite SUVR of the amyloid positive scans was higher than the mean composite SUVR of the amyloid negative scans (^18^F-florbetapir: 1.46 ± 0.18 and 0.99 ± 0.11, ^11^C-PiB: 1.62 ± 0.46 and 1.01 ± 0.13, and ^18^F-florbetaben: 1.73 ± 0.22 and 1.25 ± 0.11, respectively).

#### 2.6.2 SUVR analysis

Following the computation of composite SUVRs for the ^18^F-florbetapir test dataset, classification results were obtained using an amyloid positivity threshold of SUVR > 1.12 (Hutton et al., 2015). Similarly, classification results were obtained from the ^18^F-florbetaben SUVRs using a threshold of composite SUVR > 1.36 (Hutton et al., 2014).

Since sPAP has not been validated for ^11^C-PiB data, two regression equations were required to obtain an amyloid positivity threshold that is appropriate for both the tracer and the SUVR calculation method. Firstly, Landau et al. (2013) provided a regression equation to convert the ^11^C-PiB threshold *t*_Jagust_ from Jagust et al. (2009) into a corresponding threshold for the quantification method used by Joshi et al. (2012):

(6) $$t_{\text{Joshi}}=0.67t_{\text{Jagust}}+0.15$$

where the subscript denotes the study from which the threshold is acquired.

Hutton et al. (2015) also provided a regression equation to convert from the Joshi et al. (2012) method threshold into an equivalent unit for sPAP *t*_sPAP_:

(7) $$t_{\text{sPAP}}=0.9782t_{\text{Joshi}}+0.04264.$$

We can combine Eqs. (6), (7) to get a final equation to convert between the ^11^C-PiB threshold *t*_Jagust_ and sPAP *t*_sPAP_:

(8) tsPAP=0.97820.67tJagust+0.15+0.04264≃0.6554tJagust+0.1894.

By substituting *t*_Jagust_ = 1.465 (Jagust et al., 2009) into Eq. (8), we get an equivalent threshold for ^11^C-PiB in sPAP *t*_sPAP_ = 1.15. Consequently, following SUVR calculation in sPAP, the accuracy of amyloid status classification in ^11^C-PiB data was assessed using an amyloid positivity threshold of composite SUVR > 1.15.

### 2.7 Image intensity

We compared our method and SUVR to the machine learning method proposed by Vandenberghe et al. (2013). In that work, the authors trained a SVM on voxel intensity to classify amyloid positivity in ^18^F-flutemetamol images. Each image is a point in high-dimensional space, in which each dimension is a voxel within the brain.

To reduce the dimension of the SVM, only voxels inside the brain were used. Once all of the images were transformed into MNI space (see Section 2.2), we constructed a brain mask using the linear MNI152 T1-weighted MR template (Mazziotta et al., 2001). The mask was dilated by 2 mm to ensure that all of the registered PET brains were wholly inside the mask. Prior to using the SVM, all of the images were normalised to have zero mean and unit variance. The SVM parameters were then optimised using the same approach as in Section 2.5.3. The optimum parameters were applied to the ^18^F-florbetapir test data, as well as the ^11^C-PiB and ^18^F-florbetaben datasets. Leave-one-out testing was used to assess the ability of the intensity-based SVMs to classify amyloid status.

## 3 Results

### 3.1 3D HOG parameter optimisation

**Fig. 4:**
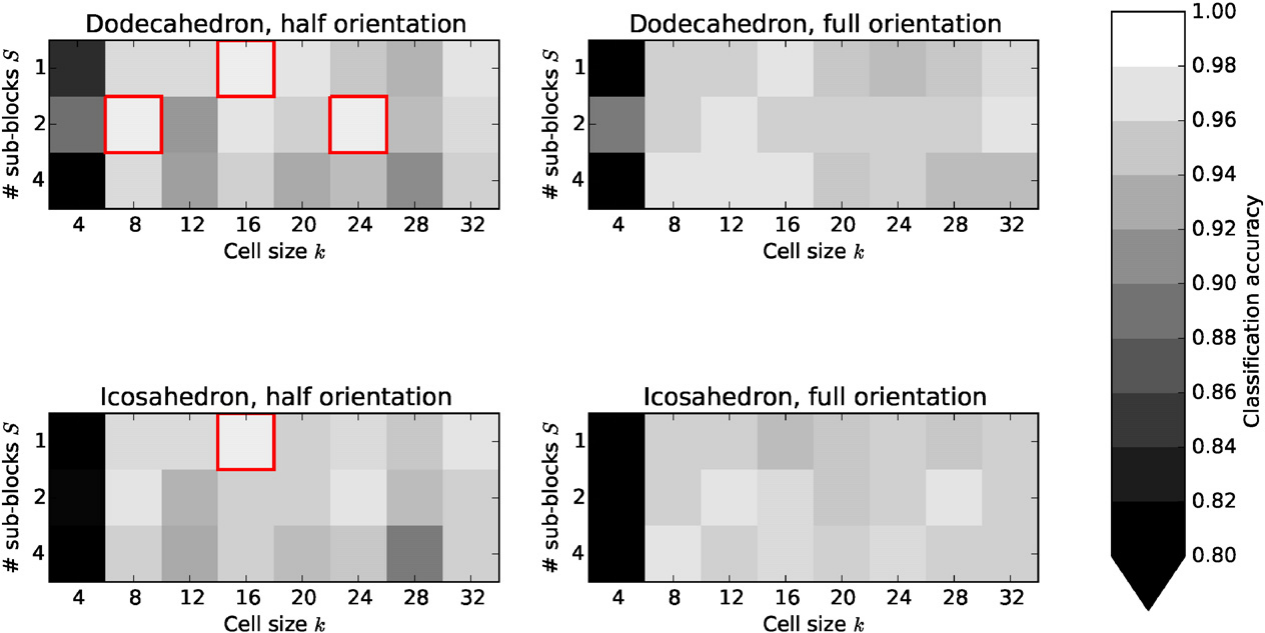
The highest classification accuracies achieved for each of the 96 3D HOG parameter combinations. Each sub-plot relates to one of the four histogram configurations (dodecahedron vs. icosahedron, and half-orientation vs. full-orientation), and shows the highest classification accuracy for each cell size *k* (horizontal axis) and number of sub-blocks *S* (vertical axis). Four parameter combinations, highlighted in red, achieved the same, highest classification accuracy (98.5%).

Fig. 4 shows the best classification accuracies achieved on the ^18^F-florbetapir training set for each of the 96 3D HOG parameter combinations. Each sub-plot relates to one of the four histogram configurations (dodecahedron vs. icosahedron, and half-orientation vs. full-orientation), and shows the highest classification accuracy for each cell size *k* (horizontal axis) and number of sub-blocks *S* (vertical axis). A cell size of *k* = 4 voxels universally resulted in the lowest classification accuracy. However, 68 different parameter combinations resulted in a mean classification accuracy greater than 95%. Four parameter combinations (highlighted in red in Fig. 4) achieved the same, highest classification accuracy of 98.5%. However, one parameter combination gave the highest combined classification accuracy, sensitivity, and specificity (98.5%, 0.973, and 1.00, respectively) on the ^18^F-florbetapir training set: cell size *k* = 16 voxels, number of sub-blocks *S* = 1, icosahedron, and half-orientation histogram. This set of optimum 3D HOG parameters was then applied to the ^18^F-florbetapir test set, and the ^11^C-PiB and ^18^F-florbetaben data.

### 3.2 Classification results

**Fig. 5:**
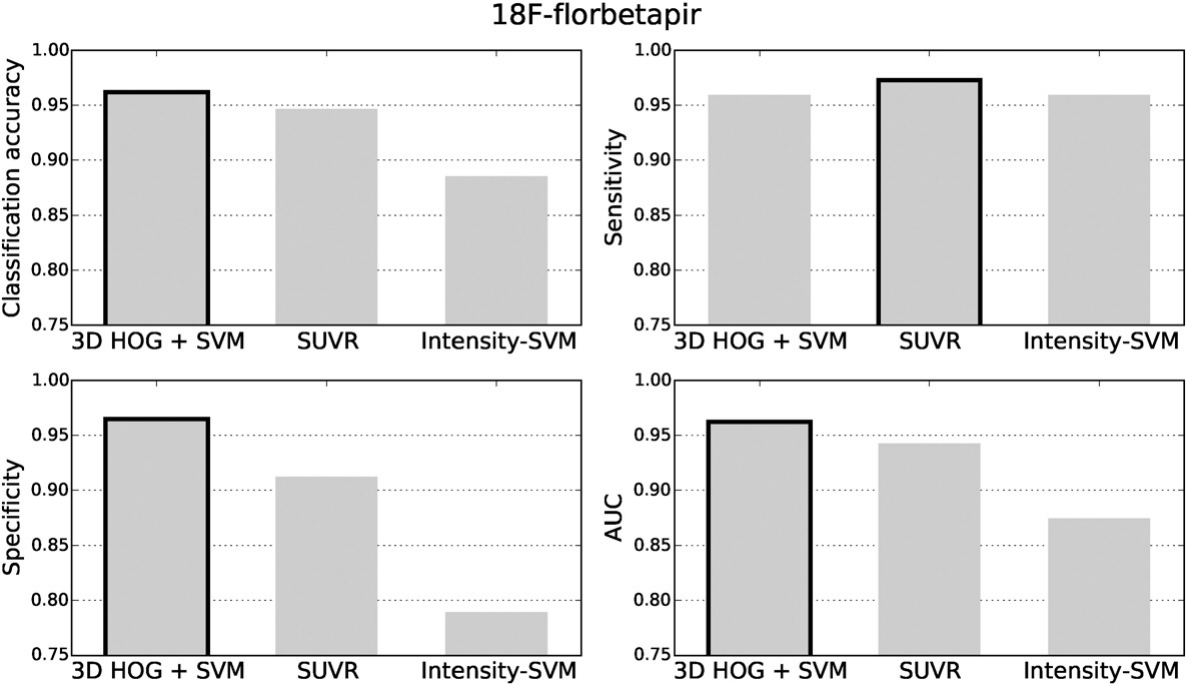
The classification accuracy, sensitivity, specificity and area under the receiver operating characteristic curve (AUC) for the ^18^F-florbetapir test data. The best results are highlighted with a black border.

**Fig. 6:**
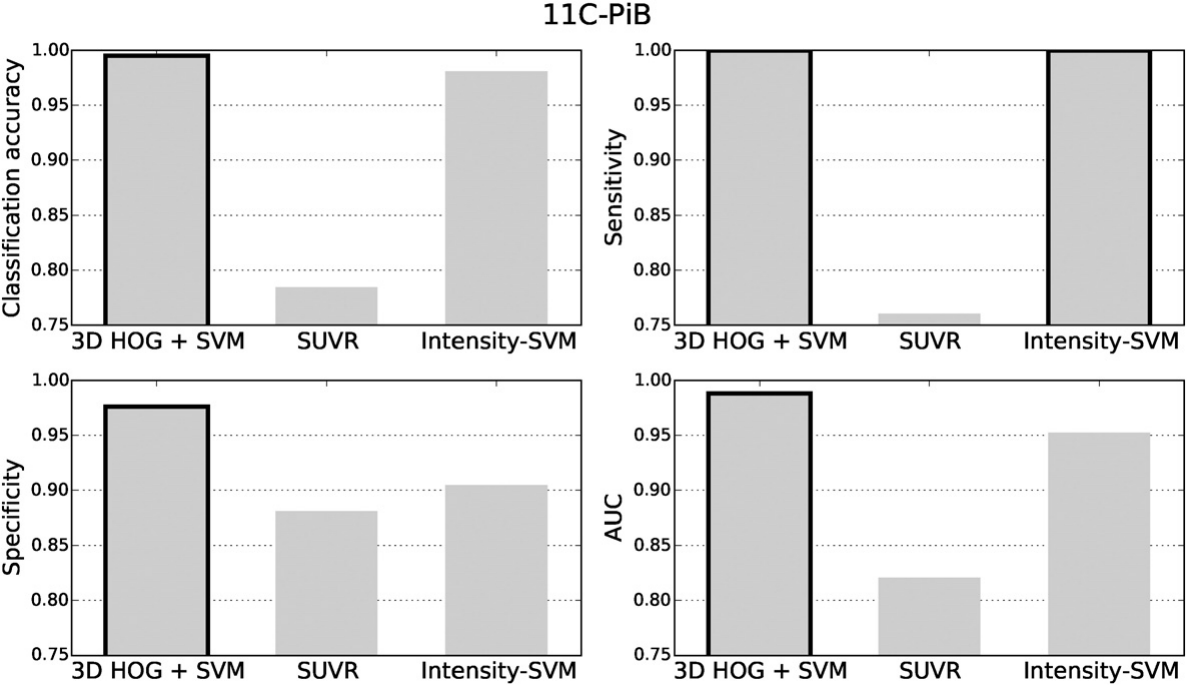
The classification accuracy, sensitivity, specificity and area under the receiver operating characteristic curve (AUC) for the ^11^C-PiB test data. The best results are highlighted with a black border.

**Fig. 7:**
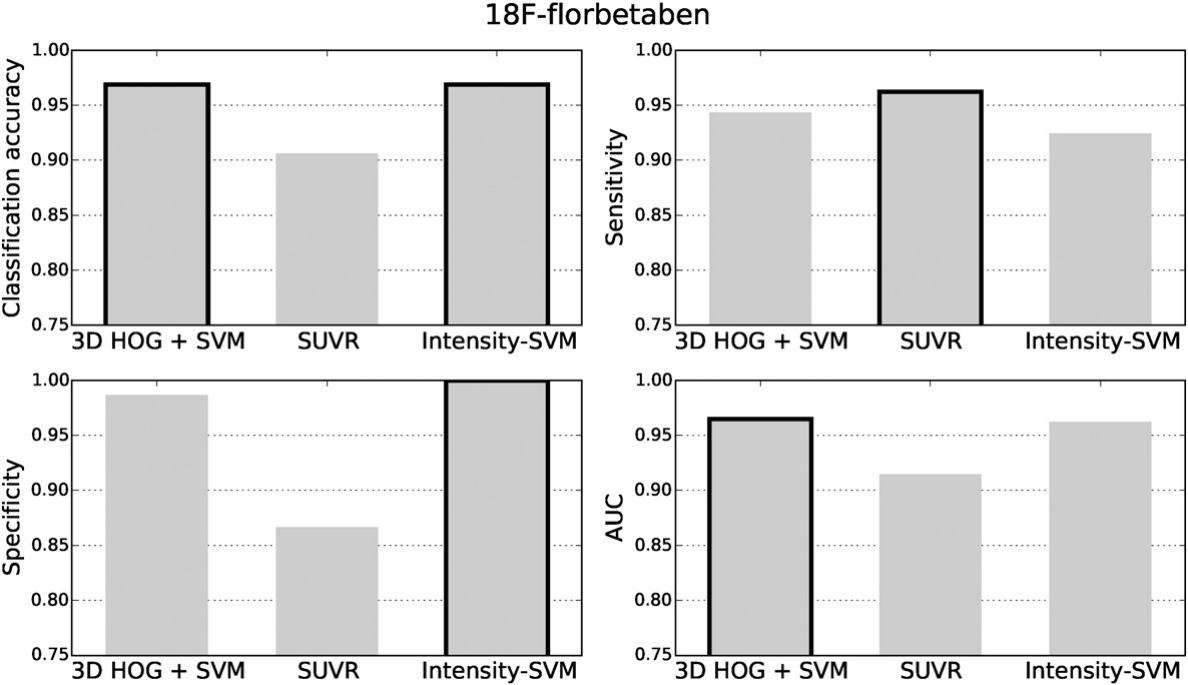
The classification accuracy, sensitivity, specificity and area under the receiver operating characteristic curve (AUC) for the ^18^F-florbetaben data. The best results are highlighted with a black border.

The classification accuracy, sensitivity, specificity and area under the receiver operating characteristic curve (AUC) for the ^18^F-florbetapir test data are shown in Fig. 5. The black borders indicate the best results. The classification results for the ^11^C-PiB and ^18^F-florbetaben datasets are presented in Fig. 6, Fig. 7, respectively. For all three tracers, the 3D HOG + SVM classification method resulted in the largest classification accuracy (96.2%, 99.5%, and 96.9%, respectively) and AUC (0.962, 0.988, and 0.965, respectively). Using the DeLong method (DeLong et al., 1988) to statistically compare the AUCs of each classification method, the 3D HOG + SVM method achieved a significantly larger AUC than the intensity-SVM method for ^18^F-florbetapir (*p* < 0.01). Furthermore, the 3D HOG + SVM classification method also achieved a significantly larger AUC than SUVR for ^11^C-PiB (*p* < 0.01). Although the 3D HOG + SVM method had a larger AUC than both the other methods for ^18^F-florbetaben, the AUCs were not significantly different. For ^18^F-florbetaben, the intensity-based SVM had the same classification accuracy as the 3D HOG + SVM method (96.9%). The 3D HOG + SVM method had a higher specificity (0.965, 0.976, and 0.987, respectively) than the SUVR method (0.912, 0.881, and 0.867, respectively) across all of the tracers tested. However, SUVR gave the highest sensitivity for ^18^F-florbetapir and ^18^F-florbetaben (0.973 and 0.962, respectively).

### 3.3 Distance to classification boundary

**Fig. 8:**
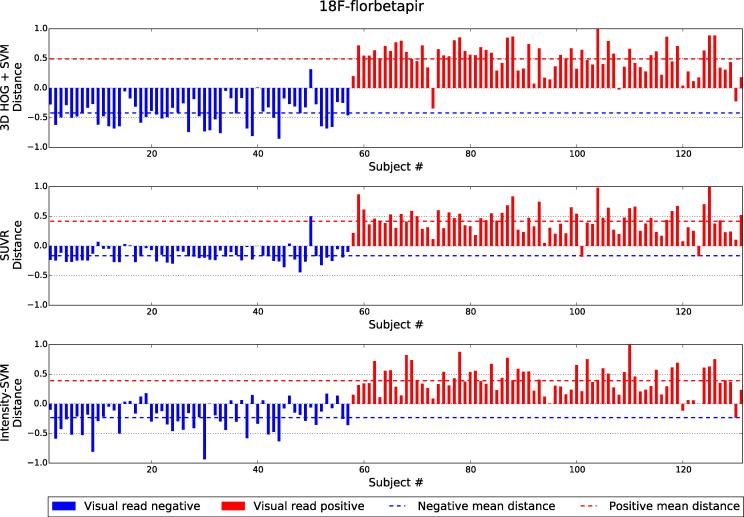
The normalised distances of ^18^F-florbetapir test subjects from the classification boundary. Subjects in blue with positive distances were incorrectly classified by the given classification method. Similarly, subjects in red with negative distances were also incorrectly classified. The dashed lines indicate the mean distance from the boundary for the subjects visually designated as amyloid positive and amyloid negative.

**Fig. 9:**
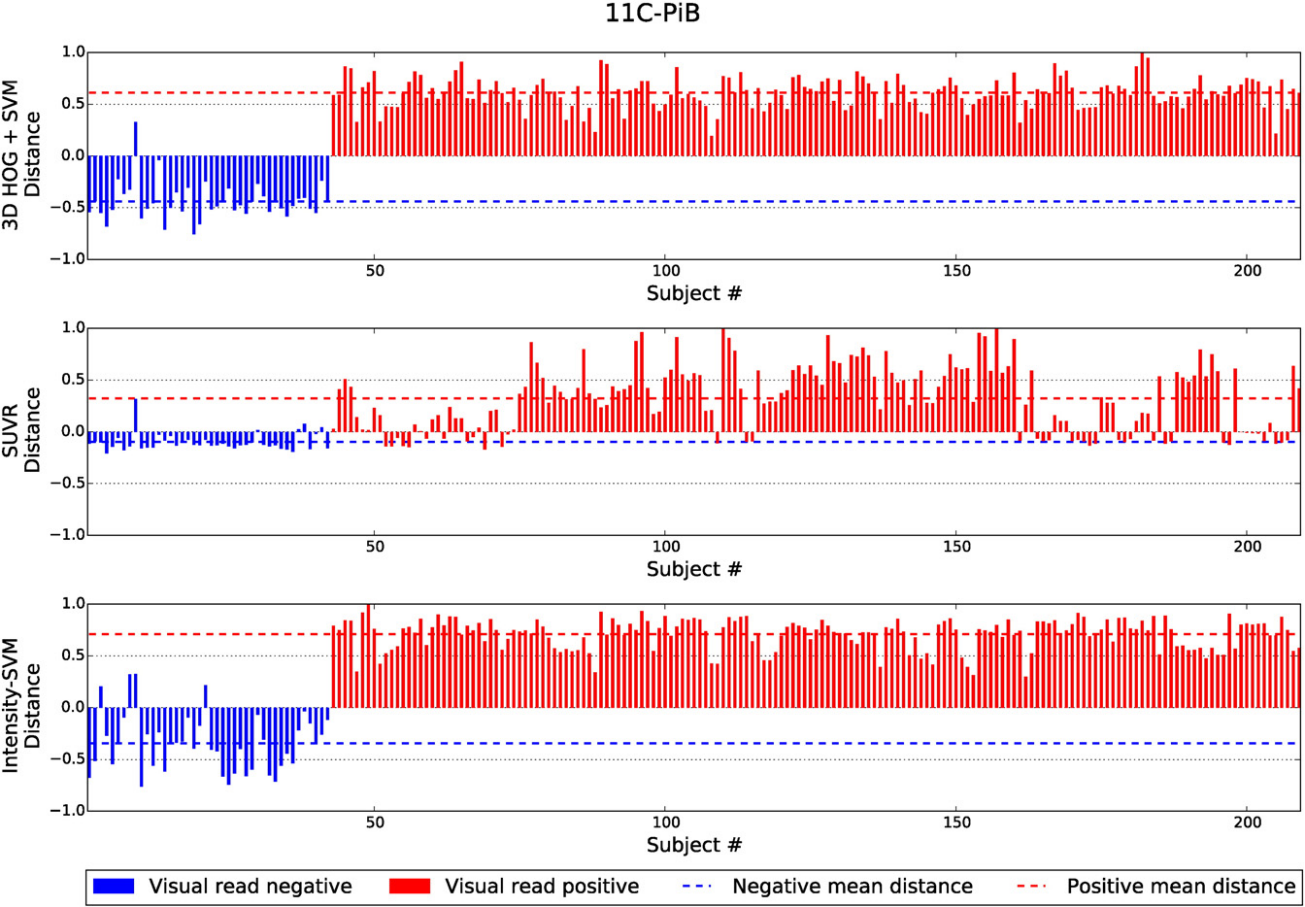
The normalised distances of ^11^C-PiB test subjects from the classification boundary. Subjects in blue with positive distances were incorrectly classified by the given classification method. Similarly, subjects in red with negative distances were also incorrectly classified. The dashed lines indicate the mean distance from the boundary for the subjects visually designated as amyloid positive and amyloid negative.

**Fig. 10:**
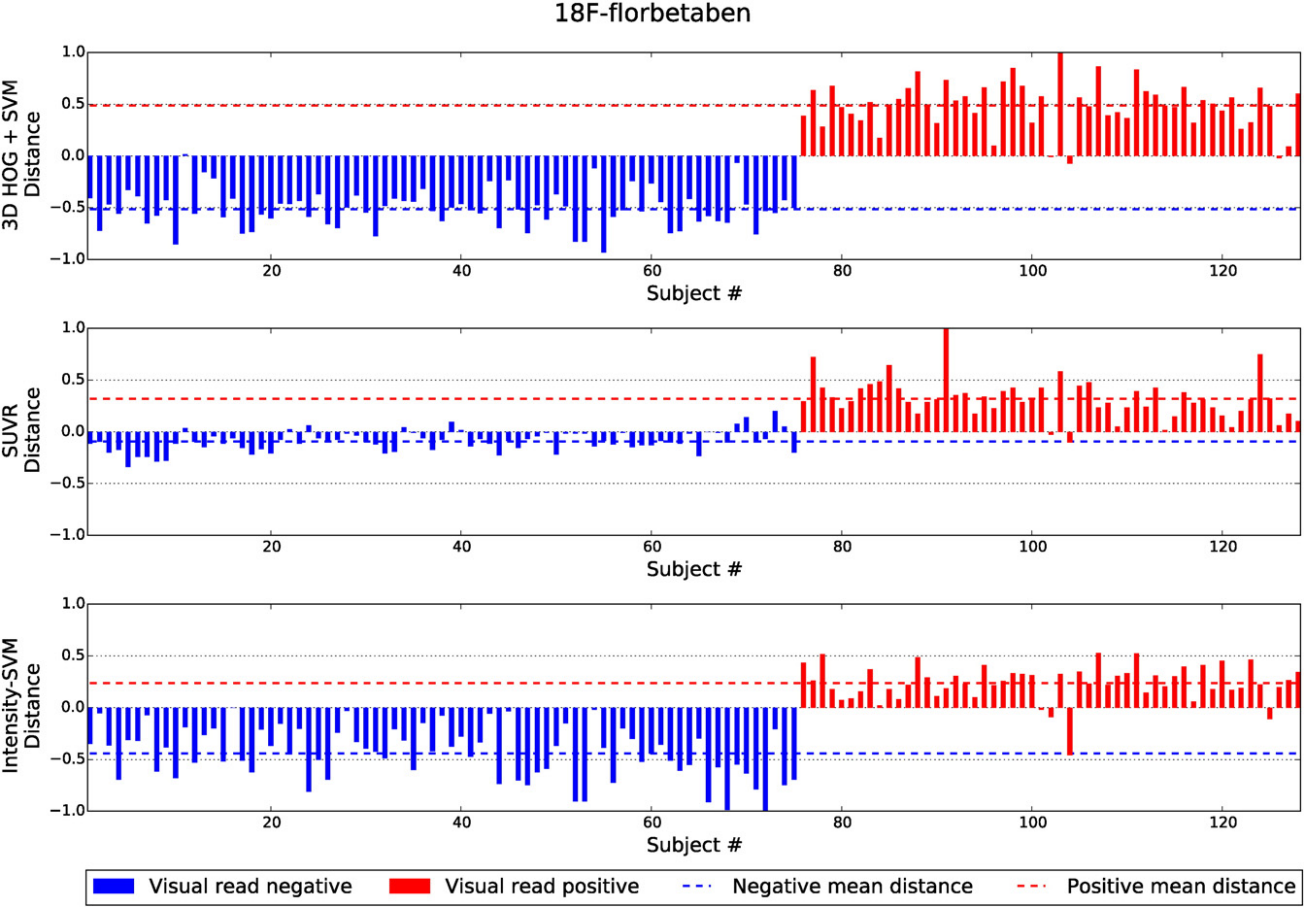
The normalised distances of ^18^F-florbetaben test subjects from the classification boundary. Subjects in blue with positive distances were incorrectly classified by the given classification method. Similarly, subjects in red with negative distances were also incorrectly classified. The dashed lines indicate the mean distance from the boundary for the subjects visually designated as amyloid positive and amyloid negative.

Fig. 8, Fig. 9, Fig. 10 show the distances of the test subjects from their respective classification boundary. For the SVM-based methods, the distances are the Euclidean distances to the decision hyperplane. For the SUVR method, the distances represent the subject's SUVR minus the threshold SUVR. The distances are normalised to the maximum absolute distance from the boundary. Smaller distances indicate a lower confidence in the final classification decision. In Fig. 8, Fig. 9, Fig. 10, subjects in blue with positive distances were incorrectly classified by the given classification method. Similarly, subjects in red with negative distances were also misclassified.

We used a two-sided *t*-test, corrected for two comparisons, to examine whether the boundary distances for the 3D HOG + SVM method were significantly greater than the other two classification methods. Values of *p* < 0.01 (*p* < 0.005, corrected) are considered significant.

Across all three tracers, the distances from the boundary for the 3D HOG + SVM method were found to be significantly greater than the distances of the SUVR method, for both amyloid positive and amyloid negative subjects. Furthermore, for both subject groups, the ^18^F-florbetapir distances are significantly greater for the 3D HOG + SVM method than the distances of the intensity-based SVM classification method. The 3D HOG + SVM distances were also significantly greater for the amyloid positive ^18^F-florbetaben subjects. In contrast, the distances for the amyloid positive ^11^C-PiB subjects were significantly greater for the intensity-based SVM method compared to the 3D HOG + SVM method.

**Fig. 11:**
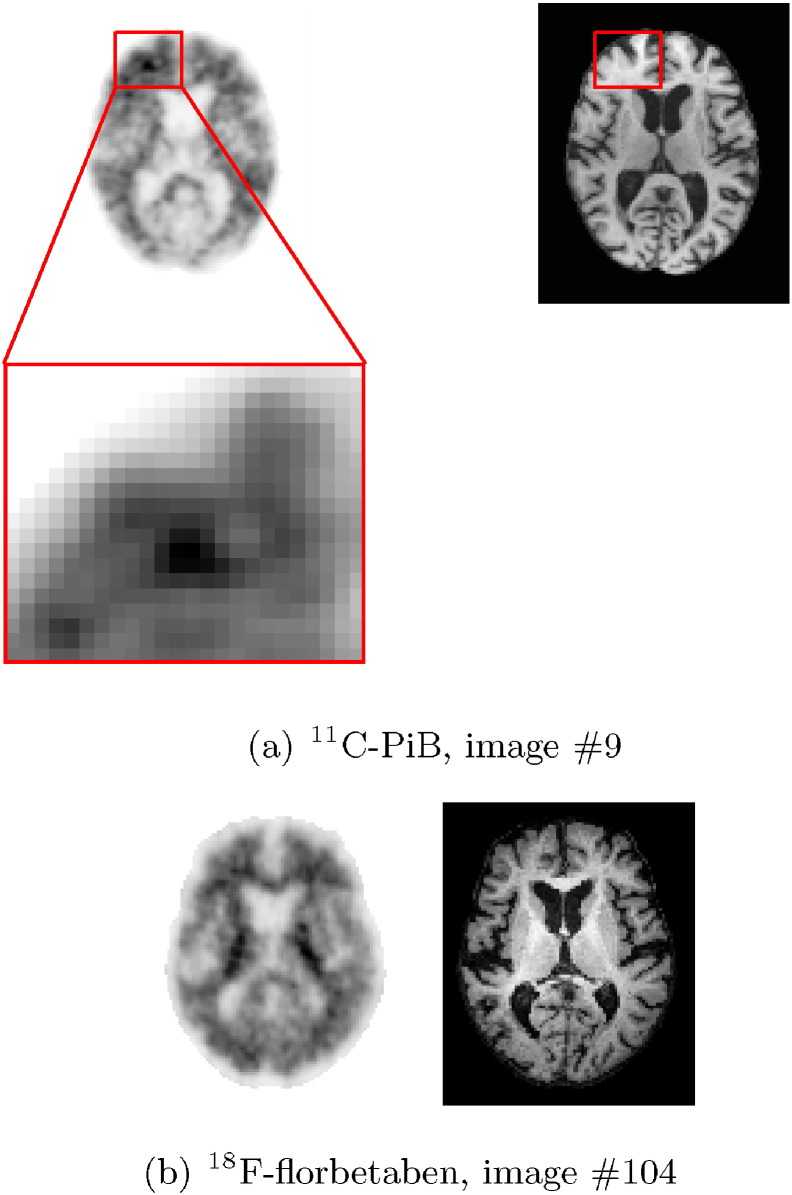
PET and MR axial slices from the two images which were classified differently to the gold standard visual assessment by all three classification methods. (a) ^11^C-PiB image #9 was visually assessed as amyloid negative, but incorrectly classified as positive. However, on closer inspection, tracer uptake was observed in the frontal region (highlighted by the red box), suggesting that the classification should be amyloid positive. (b) ^18^F-florbetaben image #104 was correctly assessed as positive, but automatically classified as amyloid negative.

It is apparent from Fig. 9 that one ^11^C-PiB image was classified differently to the gold standard visual assessment by all three classification methods (image #9). Similarly, in Fig. 10, ^18^F-florbetaben image #104 was classified differently to the gold standard across all three methods. Axial slices from the PET and MR volumes of these outliers are shown in Fig. 11.

Although scans with equivocal visual reads were eliminated from the three datasets prior to classification analysis, we computed the normalised distances of the equivocal scans from the classification boundary for each automated classification method and tracer. The 3D HOG + SVM method resulted in the largest mean absolute distance for ^18^F-florbetapir (0.418), and the intensity-based SVM achieved the largest mean absolute distance for both ^11^C-PiB and ^18^F-florbetaben (0.878 and 0.700, respectively).

## 4 Discussion

### 4.1 Classification accuracy

In this paper we have proposed an amyloid status classification method that is independent of the predefined regions of interest and amyloid positivity thresholds typically used to classify based on SUVR. Our method has been shown to generalise across multiple tracers, and could be used as an adjunct to visual interpretation of PET images, which is currently the standard method for clinical assessment. In a clinical setting, it would be straightforward to interpret the results due to the method's straightforward, binary output (amyloid positive or amyloid negative). Moreover, unlike SUVR, knowledge of the specific amyloid positivity thresholds for each tracer is not required, and there is no need to check that the target/reference regions are positioned correctly on the image.

Using visual assessment of the images as the gold standard, the 3D HOG + SVM method resulted in the highest classification accuracy and AUC for all three of the tracers we evaluated. This could be because it uses local intensity gradients as features, rather than intensity directly. Conceptually, this is similar to visual assessment of ^18^F-florbetapir, which utilises local loss of contrast between adjacent grey and white matter, and consequently, the 3D HOG + SVM method is robust to spatially varying intensity levels. This is advantageous in PET image classification, when data acquired, and reconstructed, at multiple sites and multiple scanners, may have different spatially varying intensities. Moreover, by quantising the gradients, the 3D HOG + SVM method is more robust to noise than the intensity-based SVM, which uses all of the voxels, which can include noise, as the feature vector. It may be possible to achieve a higher classification accuracy with the intensity-based SVM method by smoothing or downsampling the data to reduce the noise.

Another reason for the high classification accuracy and AUC of the 3D HOG + SVM method could be the use of cells, instead of individual voxels. As a result, 3D HOG can cope well with minor misregistration of the brain to MNI space. When calculating SUVRs, a small misalignment of the PET brain could result in tracer uptake appearing to be outside of the region of interest. As a result, this may have some effect on SUVR.

For the ^18^F-florbetapir and ^18^F-florbetaben tracers the SUVR classification method exhibited the highest sensitivity. One reason for this could be the nature of the amyloid positivity thresholds. In a clinical setting, a test with a high sensitivity will rarely misdiagnose a diseased patient. Although false positives could cause unnecessary worry or treatment, a false negative patient could miss out on vital support and care. However, this notion is merely speculative, especially given that amyloid PET studies have generally not supported a clinically relevant bias towards reporting a scan as positive, and furthermore, there is currently no effective treatment for Alzheimer's disease.

In contrast, the results in Fig. 6 show a relatively lower classification accuracy (78.5%) and sensitivity (0.760) for SUVR compared to the 3D HOG + SVM and intensity-based SVM methods in ^11^C-PiB cases. This could be due to the choice of brain regions used to compute the SUVR, as well as the amyloid positivity threshold. Since ^11^C-PiB has not been approved for clinical use, the brain regions used to compute the ^11^C-PiB SUVRs were taken from ^18^F-florbetapir literature. Moreover, the sPAP quantification software that was used to calculate the SUVRs has not been validated with ^11^C-PiB data, so we converted the amyloid positivity threshold from Jagust et al. (2009) (1.465) to the sPAP scale (1.15). Eq. (8) was constructed from two separate regression equations and implicitly assumed that the SUVR behaves linearly between the three quantification methods. In reality, this assumption may not be true. Moreover, not only could the original threshold of 1.465 be suboptimal, but every conversion introduces rounding errors. A slight change in the threshold can have an effect on the classification results. For example, by using a threshold of 1.16 instead of 1.15, the classification accuracy decreases from 78.5% to 78.0%. Similarly, the sensitivity decreases from 0.760 to 0.754. Although these differences appear small, on a large population a 0.5% difference in classification accuracy could mean substantial numbers of patients are misdiagnosed. This result highlights the need for careful validation of new SUVR computation methods, and amyloid positivity thresholds, for both existing and new tracers, which is one of the goals of the Centiloid Project (Klunk et al., 2015).

### 4.2 3D HOG parameters

The results of the 3D HOG parameter optimisation in Fig. 4 suggest that this method is likely to give high classification accuracy, even with suboptimal parameters. This is confirmed by the fact that 68 out of 96 parameter combinations resulted in a classification accuracy greater than 95%. Cell size *k* had the most profound effect on classification accuracy, so very small or very large values of *k* should not be used. Interestingly, the optimum number of subblocks was *S* = 1. This is equivalent to not using sub-blocks, and only calculating gradients in the larger cells. One possible reason for this result is that PET has a comparatively low resolution compared to the video sequences for which 3D HOG was designed. As a result, there is no need to average gradients over numerous sub-blocks. All four of the highest scoring parameter combinations used half-orientation histograms, suggesting that the sign of the gradient is uninformative in this particular application. This seems reasonable, given that the visual reading instructions for ^18^F-florbetaben state that the images should be displayed in grey scale or inverse grey scale (NeuraCeq, 2014).

### 4.3 Distance from the classification boundary

For all cases except positive ^11^C-PiB subjects, the 3D HOG + SVM method resulted in the greatest mean distances from the decision boundary. A large distance is desirable, since points near the classification boundary represent low-confidence classification decisions. Again, one reason for the superior performance of the 3D HOG + SVM method could be that it utilises image gradients, rather than direct voxel intensities. The resulting invariance to spatially varying intensity levels and noise robustness allows the two populations to be more easily separated than using SUVR or the intensity-based SVM method.

The small distance between amyloid negative subjects and the SUVR threshold supports its relatively lower specificity in Fig. 5, Fig. 6, Fig. 7. The subjects close to the threshold are classified with a lower confidence, and are more likely to be misclassified as false positives.

The two images that were classified differently to the gold standard visual assessment (^11^C-PiB image #9 and ^18^F-florbetaben image #104) were visually assessed again by two of the original three readers. Although the ^18^F-florbetaben image was again assessed to be an amyloid positive subject by the readers, the distance from the classification boundary for the SUVR and 3D HOG + SVM classification methods was small. This indicates that the different automatic classification decision has low confidence, and that this subject is a particularly difficult borderline case. After visual reassessment of the ^11^C-PiB case, for which the gold standard amyloid status was negative, a small region of tracer uptake was identified in the frontal lobe (highlighted by the red box in Fig. 11(a)), suggesting that the gold standard amyloid status may have been incorrect for this case. This highlights the importance of using an adjunct to visual assessment of amyloid images. In this study, the visual reads were conducted using PET volumes only. However, using MR images to help localise tracer uptake might make visual assessment more robust.

In this study, scans were given an “equivocal” visual assessment if they did not clearly fulfil the stringent definitions of amyloid positive or amyloid negative scans. Typically, scans were only designated as equivocal when readers lacked confidence in a final classification due to poor image quality. For this reason, equivocal scans are the type of scans for which automated classification could be most useful. Although there is no gold standard to which the classification results can be compared, the distances of the equivocal scans from the boundary indicate the level of confidence in the final classification of the automated classification methods. The 3D HOG + SVM method achieved the largest mean absolute distance from the boundary (0.418) for the equivocal ^18^F-florbetapir scans, suggesting a higher level of confidence in the final classification than the SUVR and intensity-based SVM methods. For the equivocal ^11^C-PiB and ^18^F-florbetaben scans, the intensity-based SVM method resulted in the largest mean absolute distance from the classification boundary (0.878 and 0.700, respectively). However, due to the small sample size (only five equivocal scans for both ^11^C-PiB and ^18^F-florbetaben), the distances for the intensity-based SVM method were not significantly larger (*p* < 0.01) than those achieved by the other two classification methods.

### 4.4 Methodological considerations

In this study, the gold standard for amyloid status was determined using criteria based on consistent visual assessments from three image readers. Although ADNI provides a clinical diagnosis (e.g. cognitively normal, mild cognitive impairment, Alzheimer's disease) for each subject at the time of the ^18^F-florbetapir and ^11^C-PiB scans, these diagnoses are determined using a range of clinical tests. Consequently, the visual interpretations of the scans may not correlate with the clinical diagnoses (Frey, 2015). For example, a subject with an amyloid negative scan may not be clinically diagnosed as a healthy control. Since the methods employed in this paper focus on classification of amyloid status using PET images only, the clinical diagnoses from ADNI were discarded. Moreover, the tracer manufacturer instructions state that a positive ^18^F-florbetapir scan does not establish a diagnosis of Alzheimer's disease or other cognitive disorder (Amyvid, 2012).

For this study, the gold standard amyloid status for each scan was obtained using the median rating from six visual assessments by three different image readers. The most junior reader interpreted the images three times and the most senior reader assessed the images once. Although the number of evaluations varied for each reader, this had little effect on the final gold standard visual assessments and classification experiments. For example, if the first assessment from each reader were used, such that each reader only contributed one data point per scan, the gold standard classification would change for only six ^11^C-PiB scans and two ^18^F-florbetaben scans. The median visual assessment for both ^18^F-florbetaben scans would change from amyloid negative to equivocal, thus excluding the scans from our study, and reducing the size of the dataset. Similarly, four of the ^11^C-PiB scans would be reclassified as equivocal scans. The median visual assessment of the remaining two ^11^C-PiB scans would change from amyloid negative to amyloid positive. Although the gold standard classification would change for 17 ^18^F-florbetapir scans if the first visual assessment from each reader were used, the median rating of all 17 scans would change to equivocal. Consequently, these 17 scans would have been excluded from this work, and therefore the effect on the classification accuracies presented in Section 3.2 would be minimal.

In this work, we optimised the 3D HOG parameters and SVM parameters completely independently of the test data. We optimised the 3D HOG + SVM parameters using the ^18^F-florbetapir training data, and used a leave-one-out testing approach on the test data for all three tracers. Since the 3D HOG + SVM parameters were optimised using ^18^F-florbetapir data only, the entire ^11^C-PiB and ^18^F-florbetaben datasets were used for testing. Therefore, leave-one-out testing was used so that the SVM classifier was always trained using data from the same amyloid PET tracer as the test data. However, if the SVM that is applied to the test data is trained using the original ^18^F-florbetapir training data,^4^ the classification accuracies for the 3D HOG + SVM method are only slightly lower than using leave-one-out testing (^18^F-florbetapir: 90.8%, ^11^C-PiB: 96.7%, ^18^F-florbetaben: 93.8%). In contrast, if the same approach is used to test the intensity-based SVM method, the classification accuracies are considerably lower than using leave-one-out testing (^18^F-florbetapir: 56.5%, ^11^C-PiB: 79.9%, ^18^F-florbetaben: 41.4%).

In future, to fully assess the generalisability of the 3D HOG + SVM method, we need to analyse the classification results obtained by optimising the 3D HOG parameters on other amyloid PET tracers than ^18^F-florbetapir. This is the subject of ongoing research, and for clarity, we chose not to present our results here. Nevertheless, our preliminary results indicate that the 3D HOG + SVM method can achieve a higher classification accuracy than SUVR and the intensity-based SVM method, regardless of the amyloid PET tracer used to optimise the 3D HOG parameters.

Although we used all of the ^11^C-PiB and ^18^F-florbetaben data that were available to us, the ADNI database contains many more ^18^F-florbetapir scans than were used in this work. Therefore, in future, it would be useful to test our method on a larger dataset.

Prior to computing the 3D HOG feature vectors, we affinely registered the PET volumes to MNI space to ensure that the cells generally contained the same brain regions across all subjects. We could have used a deformable registration algorithm, however, to keep the pre-processing steps of our method in line with the sPAP SUVR method, affine registration was used. Furthermore, it has been shown that classification of Alzheimer's disease patients versus cognitively normal controls using SUVR is not affected by the registration method (affine versus deformable registration) (Cattell et al., 2015).

Many other feature descriptors have been developed in addition to histograms of oriented gradients. For example, the Scale-Invariant Feature Transform (SIFT) algorithm has been successfully used for object recognition in computer vision tasks (Lowe, 1999), and has also been used in feature-based morphometry in MRI to distinguish between patients with Alzheimer's disease and healthy controls (Toews et al., 2010). Nevertheless, we chose to use 3D HOG features due to their simplicity and speed of computation. Moreover, unlike SIFT and Speeded Up Robust Features (SURF) (Bay et al., 2006), HOG operates on a dense grid of cells rather than individual points of interest. A larger set of image descriptors over a dense grid will typically offer more information than similar descriptors evaluated at a sparse set of image points.

Unlike the original intensity-based SVM method proposed by Vandenberghe et al. (2013), which used a SVM with a linear kernel, we used a SVM with a Gaussian radial basis function kernel. Our primary reason for using a non-linear kernel was because the subjects in the original input space of the SVM might not be linearly separable. Although a linear SVM is faster to compute, and non-linear kernels can give rise to overfitting, it has been shown that if complete model selection using the Gaussian kernel has been conducted, there is no need to consider a linear SVM (Keerthi and Lin, 2003). In this work, we optimised the parameters of the SVM and Gaussian kernel on the ^18^F-florbetapir training data only, using a grid search of parameters.

On a practical level, our 3D HOG + SVM method uses less memory than the intensity-based SVM method. The 3D HOG feature vector comprised 1500 elements, whereas the feature vector for the intensity-based method contained an element for each voxel inside the brain mask (290,409 elements in total). As the number of elements increases, so does the time taken to train the SVM. The SUVR is also very quick to compute, but unlike our method, knowledge of the underlying anatomy and disease pathology is required in order to choose suitable target and reference regions.

### 4.5 Conclusion

In this paper we have proposed a machine learning method for amyloid status classification based on histograms of oriented three-dimensional gradients. We compared our method to SUVRs obtained from clinically validated amyloid quantification software, as well as another machine learning method based solely on image intensity (Vandenberghe et al., 2013). Across three separate amyloid tracers, our method achieved the highest classification accuracy and area under the receiver operating characteristic curve. Unlike SUVR, our 3D HOG + SVM method required very little recalibration between tracers, and we showed that our method has the potential to produce satisfactory results even with suboptimal parameters. Moreover, the large separation between the population groups suggests that our method makes fewer low-confidence classification decisions. In addition, in the future, we plan to specify a band of indecision on either side of the classification boundary to give visual readers a measure of confidence in the automatic classification, as well as their own classification decision.

## Figures and Tables

**Table 1:** Examples of the different regions used to calculate composite SUVRs for ^11^C-PiB, ^18^F-florbetapir, and ^18^F-florbetaben. The regions and amyloid positive/negative threshold are specific to each study.

Tracer	Study	Target regions	Reference region	Threshold
^11^C-PiB	Jagust et al. (2009)	Anterior cingulate, posterior cingulate/precuneus, prefrontal, lateral temporal, parietal cortex	Cerebellar grey matter	1.465
	Jack et al. (2008)	Anterior cingulate, prefrontal, orbitofrontal, parietal, posterior cingulate/precuneus, temporal	Cerebellar grey matter	1.5
^18^F-florbetapir	Fleisher et al. (2011)	Medial orbital frontal, temporal, anterior cingulate, posterior cingulate, parietal lobe, precuneus	Cerebellum	1.17
	Joshi et al. (2012)	Frontal, temporal, parietal, anterior cingulate, posterior cingulate, precuneus	Whole cerebellum	1.10
^18^F-florbetaben	Villemagne et al. (2011)	Dorsolateral prefrontal, ventrolateral prefrontal, orbitofrontal, superior parietal, lateral temporal, lateral occipital, anterior cingulate, posterior cingulate	Cerebellar cortex	1.4
	Barthel et al. (2011)	Frontal, parietal, lateral temporal, anterior cingulate, posterior cingulate, occipital	Cerebellar cortex	1.39

**Table 2:** Demographics of the amyloid positive (P) and amyloid negative (N) subjects used in this study.

	^18^F-florbetapir		^11^C-PiB		^18^F-florbetaben	
	P	N	P	N	P	N
Count	149	115	167	42	53	75
Age ± std. dev.	75.6 ± 7.6	74.7 ± 8.5	76.7 ± 7.6	76.1 ± 7.4	72.0 ± 7.9	69.0 ± 7.0
Sex (male/female)	84/65	60/55	103/64	27/15	27/26	35/40

## Footnotes

2 http://adni.loni.usc.edu/wp-content/uploads/2008/07/adni2-procedures-manual.pdf.

3 http://www.fil.ion.ucl.ac.uk/spm/software/spm8/.

4 i.e. If a leave-one-out testing approach is not used, and the SVM used to classify the ^11^C-PiB and ^18^F-florbetaben data is trained using ^18^F-florbetapir data.
